# A Novel *Rhizobium* sp. Chiba-1 Strain Exhibits a Host Range for Nodule Symbiosis in *Lotus* Species

**DOI:** 10.1264/jsme2.ME23056

**Published:** 2023-12-01

**Authors:** Yuhei Chiba, Mao Sasaki, Sachiko Masuda, Arisa Shibata, Ken Shirasu, Yasuyuki Kawaharada

**Affiliations:** 1 United Graduate School of Agricultural Sciences, Iwate University, 3–18–8, Ueda, Morioka, Iwate 020–8550, Japan; 2 Graduate School of Arts and Sciences, Iwate University, 3–18–8 Ueda, Morioka, Iwate 020–8550, Japan; 3 RIKEN Center for Sustainable Resource Science, Yokohama, 230–0045, Japan; 4 Department of Plant BioSciences, Faculty of Agriculture, Iwate University, 3–18–8, Ueda, Morioka, 020–8550, Iwate, Japan

**Keywords:** symbiotic nodulation, host specificity, host range, rhizobium, Lotus species

## Abstract

Rhizobia are soil bacteria that induce the formation of nodules in the roots of leguminous plants for mutualistic establishment. Although the symbiotic mechanism between *Lotus japonicus* and its major symbiotic rhizobia, *Mesorhizobium loti*, has been extensively characterized, our understanding of symbiotic mechanisms, such as host specificity and host ranges, remains limited. In the present study, we isolated a novel *Rhizobium* strain capable of forming nodules on *L. burttii* from agricultural soil at Iwate prefecture in Japan. We conducted genomic and host range ana­lyses of various *Lotus* species. The results obtained revealed that the novel isolated *Rhizobium* sp. Chiba-1 was closely related to *R. leguminosarum* and had a wide host range that induced nodule development, including *L. burttii* and several *L. japonicus* wild-type accessions. However, *L. japonicus* Gifu exhibited an incompatible nodule phenotype. We also identified the formation of an epidermal infection threads that was dependent on the *Lotus* species and independent of nodule organ development. In conclusion, this newly isolated *Rhizobium* strain displays a distinct nodulation phenotype from *Lotus* species, and the results obtained herein provide novel insights into the functional mechanisms underlying host specificity and host ranges.

The mutually beneficial connection between legume plants and rhizobia is known as symbiotic nodulation. Rhizobia enter and colonize root nodules, new organs generated by the host legume plant, and convert atmospheric nitrogen into ammonium, which is then used by the host legume plant. In exchange, the host legume plant delivers carbon energy generated by photosynthesis to rhizobia (Udvardi and Poole, 2013). The capacity of these nodules to directly connect with each other through chemical signals and perception is critical to the development of this nodule symbiosis.

Legume roots produce phenolic chemicals known as flavonoids, which are recognized by rhizobia via the NodD protein in first contact for the formation of nodule symbiosis (Perret *et al.*, 2000). In rhizobia, the NodD-flavonoid complex triggers the expression of nodulation (*nod*) genes, resulting in the production and secretion of lipochitooligosaccaride molecules known as Nod factors. Once the legume plant perceives and recognizes its Nod factors through NFR1/LYK3 and NFR5/NFP LysM-RLKs, it commences nodulation processes (Amor *et al.*, 2003; Limpens *et al.*, 2003; Madsen *et al.*, 2003; Radutoiu *et al.*, 2003). Symbiotic rhizobia then invade nodules through intracellular or intercellular mechanisms that are controlled in epidermal infection thread-dependent or -independent manners (Madsen *et al.*, 2010; Montiel *et al.*, 2021; Quilbé *et al.*, 2022). These interactions depend on the ability of rhizobia and the host legume plant to perceive and respond to specific mole­cular signals from their counterparts.

These interactions also serve as the foundation for the host range and host specificity in symbiotic nodulation (Walker *et al.*, 2020). Legume plants synthesize and exude different types of flavonoids, and only a particular *Rhizobium* has the ability to recognize these flavonoids using the NodD protein (Liu and Murray, 2016). A previous study reported that when the *nodD* gene of the broad-host range *Rhizobium* sp. NGR234 was introduced into the limited-host range *R. leguminosarum* biovar *trifolii* ANU843, this strain formed symbiotic nodules on the different host plants *Trifolium* sp. and non-legume *Parasponia* (McIver *et al.*, 1989). Nod factor synthesis genes are also important for host specificity. When *nodABC* genes in *S. meliloti* GMI51 were transferred to *R. tropici* CNF299, the hybrid *R. tropici* strain more strongly induced nodules on alfalfa than *R. tropici* CNF299 (Roche *et al.*, 1996). Furthermore, the introduction of *R. meliloti* RCR2011 *nodH*, *nodEF*, and *nodQ* genes into *R. leguminosarum* bv. *trifolii* ANU843 and *R. leguminosarum* bv. *viciae* 248 changed the host plant from *T. repens* and *Vicia sativa* to *Medicago sativa* (Debellé *et al.*, 1988; Faucher *et al.*, 1989). In contrast to the wild-type strain, transconjugant *R. leguminosarum* bv. *viciae* RBL5560 containing the *nodZ* gene, given the fucosyl residue on Nod factors from *Bradyrhizobium diazoefficiens* USDA110, exhibited the ability to induce nodule formation in *M. atropurpureum*, *G. soja*, *V. unguiculata*, and *L. leucocephala *(López-Lara *et al.*, 1996). Therefore, Nod factors, which are critical for early interactions, dictate each *Rhizobium*’s host range and host specificity based on its core structure and side chains.

The rhizobial type III or IV secretion system, which injects these effector proteins directly into host plant cells, also controls host ranges and host specificity. Previous studies demonstrated that the NopP and BEL2-5 effectors of *B. diazoefficiens* USDA122 or *B. elkanii* USDA61 induced incompatibility with *G. max* carrying the *Rj2* or *Rj4* alleles (Faruque *et al.*, 2015; Sugawara *et al.*, 2018; Ratu *et al.*, 2021); these specific interactions suppressed nodule development via effector-triggered immunity (Grundy *et al.*, 2023). The msi059 effector injected into host cells through the type IV secretion system of *Mesorhizobium loti* R7A was found to inhibit nodule formation in *L. corniculatus* and *Leucaena leucocephala* (Hubber *et al.*, 2004).

Lipopolysaccharides (LPS) and exopolysaccharides (EPS) are rhizobial polysaccharides that control the host range. Several mutants of *S. meliloti* Rm1021 with changes in LPS structure showed impaired symbiosis in *M. truncatula*, but not in *M. sativa* (Campbell *et al.*, 2003). *Exo* genes from *S. meliloti* Rm1021, which are involved in the production of succinoglycans, were transferred to *S. meliloti* Rm41 forming non-nodule in *M. truncatula* A17. This led to the synthesis of similar succinoglycans and restored symbiotic nodules with them (Simsek *et al.*, 2007). The *exoU* mutant of *M. loti* R7A synthesized truncated EPS and caused symbiosis incompatibility in *L. japonicus* Gifu by recognizing the EPR3 receptor, but did not in *L. japonicus* MG20 (Kelly *et al.*, 2013; Kawaharada *et al.*, 2015, 2017, 2021; Liu *et al.*, 2018).

Several *Rhizobium* genera, including *Mesorhizobium*, *Ensifer* (*Sinorhizobium*), *Bradyrhizobium*, *Rhizobium*, and *Aminobacter*, have been isolated from *Lotus* nodules growing in natural areas (Andrews and Andrews, 2017; Lorite *et al.*, 2018). These findings suggest the ability of *Lotus* species to form nodules with many types of rhizobia as well as their wide host range; however, their host specificity remains unknown. Recent studies showed that *B. elkanii* USDA61 formed nodules on *L. burttii* and *L. japonicus* MG20, but not on *L. japonicus* Gifu, whereas the same genus as *Bradyrhizobium* sp. SUTN9-2 formed nodules on *L. burttii* and *L. japonicus* Gifu, but not on *L. japonicus* MG20 depending on the type III secretion system (Hashimoto *et al.*, 2020; Kusakabe *et al.*, 2020). Another genus, *S. fredii* HH103, also exhibits a different nodule phenotype among *Lotus* species. *L. burttii* inoculated with *S. fredii* HH103 formed mature nodules through the intercellular infection process, whereas *L. japonicus* Gifu did not. These phenotypes were found to depend on the NopC effector injected by the type III secretion system (Acosta-Jurado *et al.*, 2016, 2019, 2020; Jiménez-Guerrero *et al.*, 2020). *R. leguminosarum* Norway isolated from *L. corniculatus* nodules has diverse nodule phenotypes in *Lotus* species (Gossmann *et al.*, 2012). *R. leguminosarum* Norway induced nodule formation on *L. burttii* and invaded it by an intercellular infection mechanism, but did not induce nodules on *L. japonicus* Gifu (Liang *et al.*, 2019). However, the host range and mode of invasion of the same *Rhizobium* genus into *Lotus* species remain unknown. In the present study, we identified the new *Rhizobium* sp. Chiba-1, which induced nodule formation on *L. burttii* and several *L. japonicus* MG accession lines, but not on *L. japonicus* Gifu or MG20. We discovered two types of epidermis-invading systems in *Rhizobium* sp. Chiba-1: epidermal infection threads and other systems that differ according to the host *Lotus* species.

## Materials and Methods

### Growth bacterial strain

*Rhizobium* and *Mesorhizobium* strains were cultured at 28°C in Tryptone Yeast medium (Beringer, 1974), while *Escherichia coli* was cultured at 37°C in Luria Bertani medium (Kawaharada *et al.*, 2007). Antibiotics at the indicated concentrations were added to culture media when necessary: fosfomycin (100‍ ‍μg mL^–1^), tetracycline (2–10‍ ‍μg mL^–1^), streptomycin (250‍ ‍μg mL^–1^), gentamycin (5 or 50‍ ‍μg mL^–1^), and chloramphenicol (20‍ ‍μg mL^–1^), for both *Rhizobium* and *Escherichia* strains.

### Nodulation assay

Seeds of *Lotus* species were sterilized using 2.4% sodium hypochlorite for 10‍ ‍min, followed by seed coat scratching with sandpaper. After 3 days, seedlings were transferred onto filter paper (No. 4A; Advantech) on modified Long Ashton medium (Kawaharada *et al.*, 2021) containing 1.4% agar slanted in 11×11‍ ‍cm Petri dishes. Each plate was inoculated 2 days later with 1‍ ‍mL of the rhizobia culture at OD_600_=0.04. Plants were grown at 23°C under a 16-h light/8-h dark cycle. Regarding *T. repens* (KANEKO SEEDS), 3-day-old seedlings from sterilized seeds were grown on 16-fold diluted modified Long Ashton medium.

### Nodule complementation assay

Co-inoculation experiments were conducted to assess the potential complementation of the nod factor-deficient phenotype in the *M. loti ∆nodAC* mutant. A 1-mL mixture of cultures consisting of the *M. loti ∆nodAC* mutant (ML101) (Takeda *et al.*, 2005) carrying pFAJGFP (Kelly *et al.*, 2013) and *Rhizobium* sp. Chiba-1 carrying pFAJDsRed (Kelly *et al.*, 2013) was prepared at a ratio of 1:1, with each culture having an OD_600_=0.02. The mixture was then inoculated onto the roots of *L. japonicus* Gifu plants. Nodules were observed 3–6‍ ‍weeks post-inoculation under a Leica M165 FC microscope.

### Microscopic observations of infection thread formation

*Lotus* sp. roots were sonicated for 30‍ ‍s for 7 to 10 days after the inoculation with *Rhizobium* sp. Chiba-1 harboring pFAJDsRed. Roots were examined under Olympus BX53 and Leica M165 FC microscopes. The CellSens Standard program (Olympus) was used to merge images of bright and DsRed fluorescence.

### GUS staining

Roots from *L. japonicus* *Nin* promoter::*GUS* and *Cbp1* promoter::*GUS* (T90) transgenic lines were analyzed in the present study. After the inoculation with *M. loti* MAFF303099 or *Rhizobium* sp. Chiba-1, roots were stained for ß-glucuronidase activity using GUS staining buffer. Staining buffer contained 0.5‍ ‍mg mL^–1^ X-Gluc, 100‍ ‍mM phosphate buffer (pH 7.0), 10‍ ‍mM EDTA, 1‍ ‍mM K_4_(Fe[CN]_6_), 1‍ ‍mM K_3_(Fe[CN]_6_), and 0.1% Triton X-100. Stained root samples were observed under the Leica M165 FC microscope. The staining process was performed at room temperature overnight.

### Phylogenetic ana­lysis

Phylogenetic trees for the *16S rRNA* and *nodC* genes were constructed using the maximum likelihood method with MEGAX software (Tamura *et al.*, 2021). The robustness of tree branchings was estimated using 1,000 bootstrap replicates. Phylogenetic trees were drawn using OIPH-PhyNE (https://www.iph.osaka.jp/s007/1000/20211129/20211129191934.html). Sequences used in phylogenetic ana­lyses were obtained from NCBI (NR_118339.1, NR_044112.1, AY509899.1, CP000133.1, JX855169.1, EF035074.2, AB680662.1, FR870231.1, NR_041396.1, NZ_CP025012.1, AB680660.1, BA000012.4, AB231916.1, AY260145.1, AF271637.1, NR_113675.1, NZ_DF820425.1, CP001191.1, NC_020059.1, JQ795195.1, FJ596038.1, KC608575.1, EF209422.1, NZ_WISX01000115.1, BA000012.4, AF217268.1, HM441255.1, DQ060002.2, NC_022536.1, NZ_CP025015.1, NC_020061.1, NC_011368.1, NZ_DF820426.1, and AP013103.1). Homology and identity searches for genes and proteins were performed using local blasts.

### *Rhizobium* sp. Chiba-1 genome sequence

Genomic DNA was extracted using phenol-chloroform. Regarding SMRTbell library preparation, genomic DNA was fragmented at 20‍ ‍kb using Megaruptor 2 (Diagenode), and the library was constructed using SMRTbell express template prep kit 2.0 according to the manufacturer’s protocol (Pacific Biosciences). Barcodes were attached to each fragmented genome, and samples were pooled and cut off at 15‍ ‍kb using the BluePippin size selection system (Sage Science). The genomic library was sequenced on a single PacBio sequel II system 2.0 cell. Reads were assembled using HGAP4 through SMRTlink (v 8.0.0), and the circularity of each contig was calculated with Circlator v 1.5.5 (Hunt *et al.*, 2015). The DDBJ Fast Annotation and Submission Tool was used to annotate genes (Tanizawa *et al.*, 2018). Sequence data were stored in AP028250 to AP028255.

## Results

### Comparable gene ana­lysis of isolated rhizobia-forming root nodules in *Lotus*

**Fig. 1.**
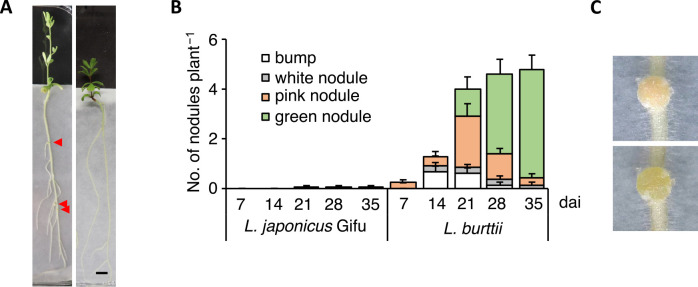
*Lotus* nodulation phenotypes inoculated with *Rhizobium* sp. Chiba-1. (A) Images of nodulation phenotypes in *L. burttii* (left) and *L. japonicus* Gifu (right) 21 days after the inoculation with *Rhizobium* sp. Chiba-1. The scale bar represents 0.5‍ ‍cm. Red arrows indicate mature-like nodules. (B) Average numbers of each nodule type formed over time (days after the inoculation) with *Rhizobium* sp. Chiba-1 in *L. japonicus* Gifu or *L. burttii*. *n*=17 for *L. japonicus* and 13 for *L. burttii*. Error bars represent standard errors (SE). (C) Nodule senescence in *L. burttii*. The upper panel shows a nodule 14 days after the inoculation, and the bottom panel shows the same nodule 22 days after the inoculation.

To identify new rhizobia noduled with *Lotus* species, we collected various soil samples from agricultural and fruit cultivation areas at Iwate University in Japan (39°42′54.2″ N, 141°08′01.0″ E). When *L. japonicus* Gifu and *L. burttii* plants were grown in these mixed soils, one nodule formed in *L. burttii* after 5‍ ‍weeks. The *Rhizobium* isolated from this nodule was named Chiba-1 strain and was reinoculated into both *L. japonicus* Gifu and *L. burttii*. Pink nodules were observed on *L. burttii*, but not on *L. japonicus* Gifu 3‍ ‍weeks after the inoculation of *Rhizobium* sp. Chiba-1 strain (Fig. 1).

**Fig. 2.**
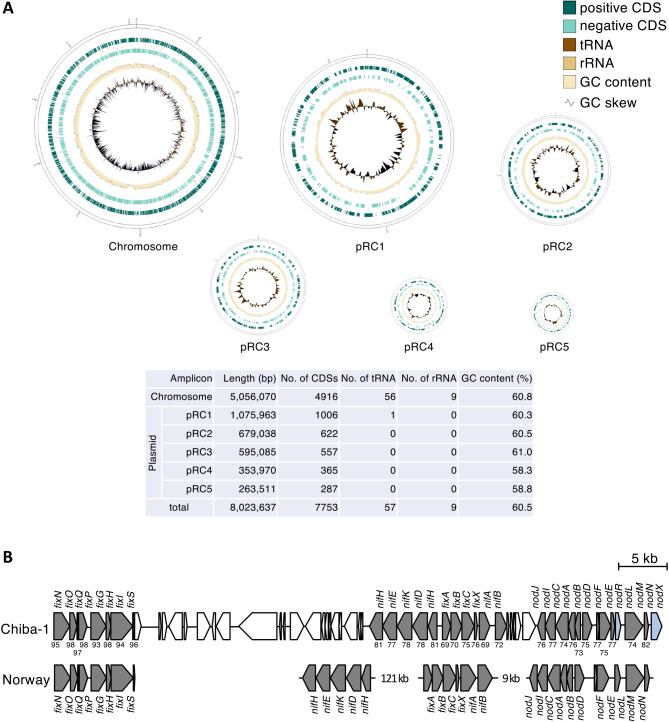
Genome structure of *Rhizobium* sp. Chiba-1. (A) The chromosome and five plasmids of *Rhizobium* sp. Chiba-1. Plasmids were drawn at four times the scale of the chromosome. Contig lengths, positive and negative strand coding sequences, tRNA, rRNA, the GC content, and GC skew were displayed from outside of the circular genome. These circular genomes were generated using Genovi (Cumsille *et al.*, 2023). (B) Comparison of symbiotic nodulation gene clusters between *Rhizobium* sp. Chiba-1 and *R. leguminosarum* Norway. Gray arrows indicate symbiotic genes, while blue arrows indicate symbiotic genes in the absence of *R. leguminosarum* Norway. Numbers between gene clusters indicate percent homology between each gene.

**Fig. 3.**
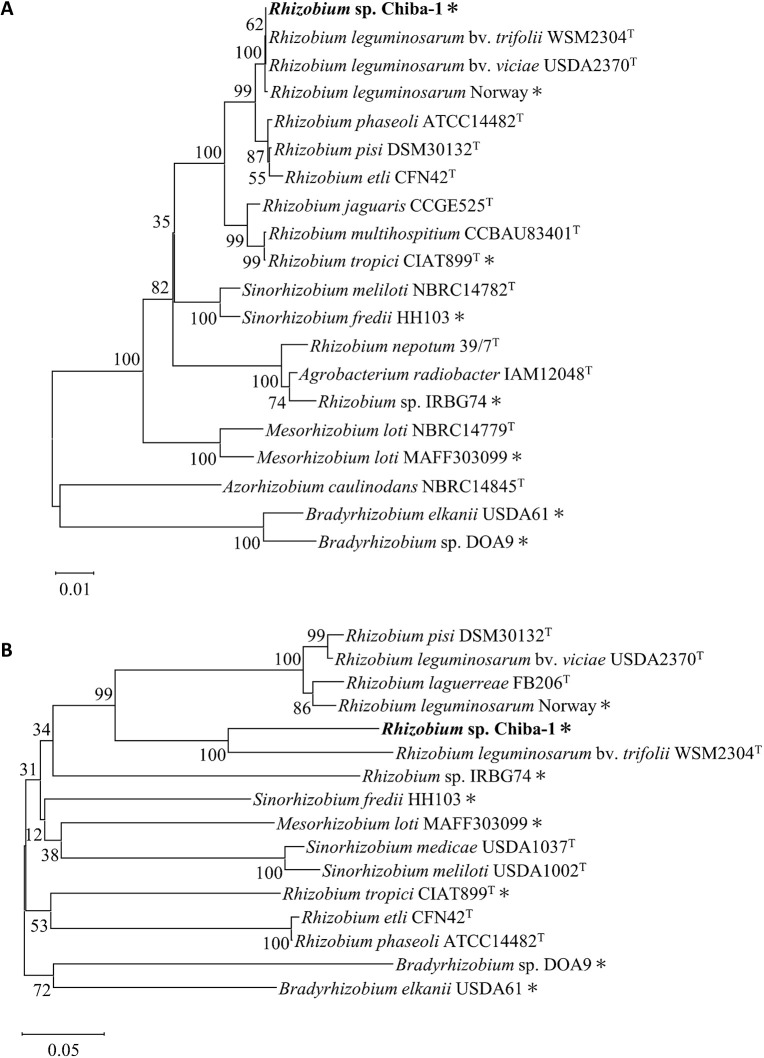
Phylogenetic trees of *16S rRNA* and *nodC* genes. The *16S rRNA* and *nodC* genes were obtained from isolated strains and their related strains that formed symbiotic nodules on legume plants. (A) A neighbor-joining phylogenetic tree of the *16S rRNA* gene was constructed using 1,362-bp partial nucleotide sequences. (B) A neighbor-joining phylogenetic tree of the *nodC* gene was constructed using 825-bp partial nucleotide sequences. Numbers at the nodes represent bootstrap values based on 1,000 replicates. The substitution rate was estimated at 0.01 and 0.05 substitutions per nucleotide position. Asterisks indicate strains that were identified to form nodules on *Lotus* species.

Whole genome sequencing by PacBio Sequel II revealed that *Rhizobium* sp. Chiba-1 consisted of one chromosome and five plasmids with 7,819 annotated genes (Fig. 2A). Based on a *16S rRNA* gene sequence ana­lysis, *Rhizobium* sp. Chiba-1 was the most closely related to *R. leguminosarum* bv. *viciae* strain USDA2370^T^ and *R. leguminosarum* bv. *trifolii* WSM2304^T^ with 100% identity. *R. leguminosarum* Norway, which had 99.9% identity to the *16S rRNA* gene of *Rhizobium* sp. Chiba-1, was previously shown to induce root nodules in *L. burttii*, but not in *L. japonicus* Gifu with similar phenotypes to *Rhizobium* sp. Chiba-1 (Fig. 3A and Supplemental Table S1) (Gossmann *et al.*, 2012). We then compared the genes in the two strains. *atpD*, *recA*, *dnaK*, and *rpoB*, four housekeeping genes from *Rhizobium* sp. Chiba-1 and *R. leguminosarum* Norway, exhibited strong homology of 93 to 99%. However, symbiotic-related genes involved in the transcription factor *nodD*, Nod factor backbone synthesis *nodABC*, adding side chains to Nod factor backbone *nodEFIJLMN* and nitrogenase *nifABDEHKN* in these strains, showed low homology of 69 to 82%, while nitrogen fixation regulator and cytochrome oxidase genes *fixABCGHINOPQSX* showed broad homology of 69 to 98% (Supplemental Table S1). Genes involved in EPS synthesis and the type IV secretion system (but not the type III secretion system), which are important for symbiotic interactions, were also identified in the *Rhizobium* sp. Chiba-1 genome (Supplemental Table S1). Low homology was noted between each symbiotic gene among rhizobia that caused nodule development in *Lotus* species when housekeeping genes and symbiotic genes were compared. Additionally, *16S rRNA* and *nodC* gene comparisons, which included a number of rhizobia-forming nodules in *Lotus* species, failed to link these genes to symbiotic relationships (Fig. 3). Based on these results, the traits of nodule phenotypes did not appear to correlate with crucial genes for the development of symbiosis.

### Host range of *Rhizobium* sp. Chiba-1 strain inducing nodule organogenesis in Lotus species

Some rhizobia species of the genera *Rhizobium* and *Bradyrhizobium* are known to exhibit nodule formation phenotypes with several *Lotus* wild-type accessions (Schumpp *et al.*, 2009; Gossmann *et al.*, 2012; del Cerro *et al.*, 2015; Acosta-Jurado *et al.*, 2016; Hashimoto *et al.*, 2020; Kusakabe *et al.*, 2020; Montiel *et al.*, 2021). Therefore, we investigated the mechanisms by which *Rhizobium* sp. Chiba-1 induced root nodule formation in *Lotus* species. *Rhizobium* sp. Chiba-1 was inoculated onto *L. burttii* in our experimental system, and mature-like pink nodules were observed after 7 days, while approximately 2 mature-like nodules per plant had formed after 21 days (Fig. 1B). However, the growth promotion of plants that formed mature-like nodules was not confirmed. At the same time point, senescence nodules, which had greened from mature-like nodules, were also noted, and approximately 4 senescent nodules per plant had formed by 35 days after the inoculation (Fig. 1C). In contrast, nodule formation was not detected on *L. japonicus* Gifu, except for one white nodule in one plant 21 days after the inoculation. Similarly, neither *L. filicaulis* nor *L. krylovii* collected from China or Kazakhstan formed nodules (Table 1 and Supplemental Fig. S1). Therefore, we selected a number of *L. japonicus* wild-type accessions procured from various locations around Japan to assess the extent of nodule development among *L. japonicus* ecotype lines (Hashiguchi *et al.*, 2011). Mature-like pink nodules including senescence green nodules were observed in less than 10% of plants in MG20, MG022, MG064, MG073, MG098, MG115, and MG122 35 days after the inoculation. No nodules were observed in any plants in MG20, MG064, or MG098. On the other hand, mature-like pink nodules were noted in the majority of plants in MG051, MG063, MG080, and MG119. Other MG accession plants showed an intermediate nodule phenotype (Table 1 and Supplemental Fig. S1). The present results revealed that the host range of *Rhizobium* sp. Chiba-1 included both *L. japonicus* intra-species and Lotus inter-species hosts. To investigate whether *Rhizobium* sp. Chiba-1 retained its nitrogen fixation ability in nodules, we inoculated *Rhizobium* sp. Chiba-1 onto *T. repens*, the main symbiotic partner of *R. leguminosarum*. Effective nodules were observed 10 days after the inoculation, one plant formed approximately 13 nodules, and nitrogen starvation in plants was eliminated 35 days after the inoculation (Supplemental Fig. S2).

### Responses of *L. japonicus* Gifu inoculated with *Rhizobium* sp. Chiba-1

**Fig. 4.**
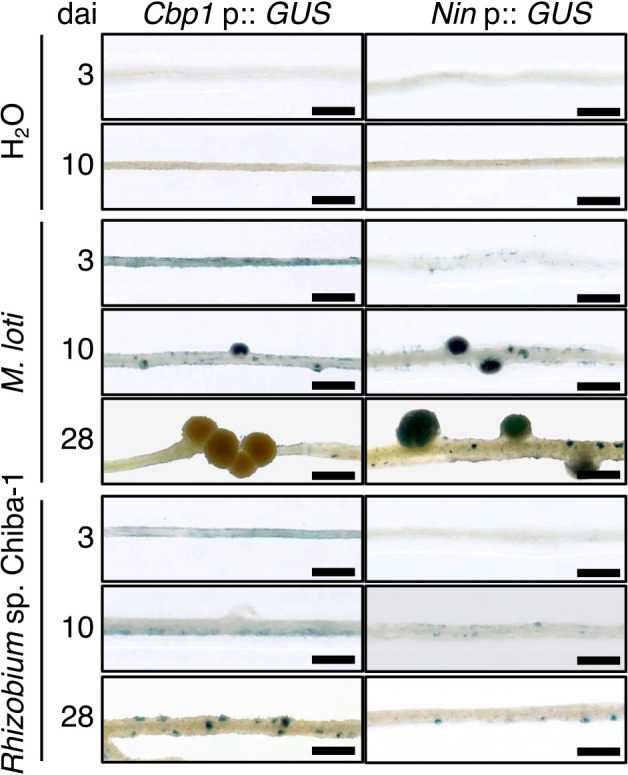
β-Glucuronidase expression induced by *Cbp1* or *Nin* promoters. *Cbp1* promoters:: *Gus* and *Nin* promoters:: *Gus* transgenic lines in *Lotus japonicus* Gifu were inoculated with *M. loti* MAFF303099, *Rhizobium* sp. Chiba-1, or H_2_O. After 3 to 28 days, roots were stained with 5-bromo-4-chloro-3-indolyl-β-D-glucuronide (X-gluc). Scale bars indicate 1‍ ‍mm.

*Rhizobium* sp. Chiba-1 did not exhibit the ability to induce nodules in *L. japonicus* Gifu. To investigate the initial response of *L. japonicus* Gifu, transgenic lines carrying the ß-glucuronidase (*Gus*) gene driven by *Nin* or *Cbp1* promoters were examined (Webb *et al.*, 2000; Radutoiu *et al.*, 2003). *M. loti* MAFF3030399 strongly induced the expression of both *Cbp1* and *Nin* 3 days after the inoculation, whereas *Rhizobium* sp. Chiba-1 only weakly induced that of *Cbp1* in the epidermis (Fig. 4). However, more than 10 days after the inoculation, *Rhizobium* sp. Chiba-1 induced *Nin* expression in the epidermis. Interestingly, 28 days after the inoculation of *Rhizobium* sp. Chiba-1, *Cbp1* and *Nin* both showed high expression in a spotted pattern in cortical cells (Fig. 4).

Although *Rhizobium* sp. Chiba-1 is capable of inducing the expression of the early symbiotic genes necessary for nodule development, we conducted co-inoculation experiments to establish whether it complemented the phenotype of the *M. loti ∆nodAC* mutant (ML101) through the Nod factors produced by *Rhizobium* sp. Chiba-1. While the *M. loti ∆nodAC* mutant alone did not induce nodule formation, a co-inoculation with both *Rhizobium* sp. Chiba-1 and the *M. loti ∆nodAC* mutant (ML101) resulted in the same nodule phenotypes as the single inoculation of *Rhizobium* sp. Chiba-1. However, no mature nodules were observed on 20 plants 35 days after the inoculation.

### Epidermal infection thread formation on *L. burttii*, but not *L. japonicus*

**Fig. 5.**
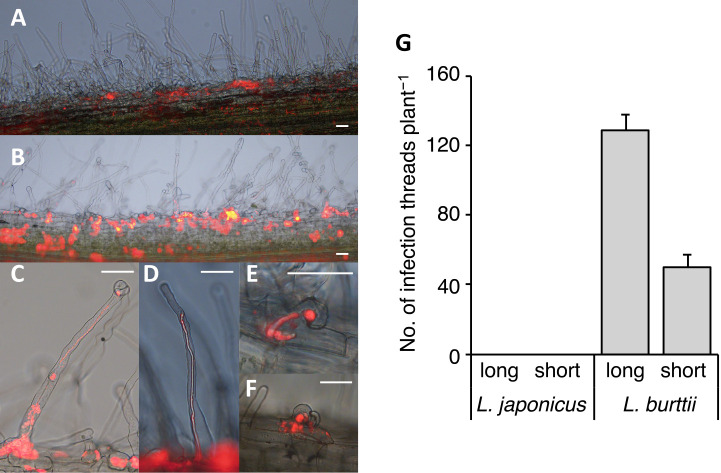
Epidermal infection thread formation. *Lotus japonicus* Gifu (A) or *L. burttii* (B, C, D, E, and F) 10 days after the inoculation with *Rhizobium* sp. Chiba-1 carrying pFAJDsRed. C and D show long infection threads, and E and F show short infection threads in *L. burttii*. Scale bars indicate 50‍ ‍μm. (G) Average number of each epidermal infection thread 10 days after the inoculation with *Rhizobium* sp. Chiba-1 carrying pFAJDsRed. *n*=10.

Nodule organ development on *L. burttii* was induced by‍ ‍*R. leguminosarum* Norway, which entered nodules via epidermal gaps between host cells (a process known as intercellular infection) in the absence of root hair‍ ‍in­fection‍ ‍threads (Liang *et al.*, 2019). In contrast, *M. loti* MAFF303099 infiltrated nodules through the development of intracellular root hair infection threads. The invasion process of *Rhizobium* sp. Chiba-1 into *Lotus* species was examined under a fluorescent microscope using a fluo­rescent strain harboring pFAJDsRed. Epidermal‍ ‍in­fection‍ ‍threads harboring *Rhizobium* sp. Chiba-1 carrying pFAJDsRed were observed in root hairs 7 to 10 days after the inoculation into *L. burttii* (Fig. 5B). We classified two types of infection threads: long thin threads and short thick threads (Fig. 5C, D, E, and F). Many of the long infection threads formed from the middle of the root hair without curling of the root hair tip, while all of the short infection threads formed through root hair curling (Fig. 5C, D, E, and F). In addition, we noted a number of irregularly broken, halfway infected threads in root hairs (Fig. 5C and F). The total number of infection threads of *L. burttii* included approximately 129 long infection threads and 50 short infection threads 10 days after the inoculation with *Rhizobium* sp. Chiba-1 (Fig. 5G). *Rhizobium* sp. Chiba-1 was attached to the root surface of *L. japonicus* Gifu, and infection threads were not observed (Fig. 5A). Additionally, no infection threads were produced by *L. japonicus* MG accessions that did or did not develop mature-like nodules (Table 1). These results show that in the symbiotic relationship between *Rhizobium* sp. Chiba-1 and *Lotus* species, infection thread development and nodule formation were separate events.

## Discussion

Legume-rhizobia symbiosis exhibits the complex and varied characteristics of host specificity and host ranges, and the underlying mole­cular pathways have yet to be examined in detail. In the present study, we isolated a novel *Rhizobium* sp. Chiba-1 from nodules in *L. burttii*, identified the host range in *Lotus* species, and elucidated the mechanisms of invasion into nodules.

*Rhizobium* sp. Chiba-1, which has a high degree of similarity with *R. leguminosarum* Norway on the *16S rRNA* gene, was discovered and exhibited host selectivity that was more similar to *Lotus* species than to *R. leguminosarum* Norway (Fig. 1 and Supplemental Table S1) (Gossmann *et al.*, 2012). In addition, compared with symbiotic genes related to Nod factor synthesis and nitrogen fixation in each pRLN3 (557,386 bp) of *R. leguminosarum* Norway and pRC4 (353,970 bp) of *Rhizobium* sp. Chiba-1, these were widely different from one another (Liang *et al.*, 2018) (Fig. 2B and Supplemental Table S1), suggesting that the genetic factors affecting the host range did not depend on the acquired symbiotic plasmid or main mole­cular cues for symbiosis. The diversity of results from phylogenetic ana­lysis of *16S rRNA* and *nodC* genes (Fig. 3) made it difficult to determine the host range of *Lotus* species.

*M. loti*, a major symbiotic partner of *L. japonicus* and *L. burttii*, forms elongated infection threads in root hairs and invades nodules (Acosta-Jurado *et al.*, 2016). In contrast, *B. elkanii* USDA61, *Rhizobium* sp. IRBG74, *R. leguminosarum* Norway, *S. fredii* HH103, *Rhizobium* sp. NGR234, and *R. tropici* CIAT899, which exhibit minor nodulation with *Lotus* species, employ two distinct epidermal infection strategies. *B. elkanii* USDA61, *Rhizobium* sp. IRBG74, and *R. leguminosarum* Norway are unable to form infection threads in root hairs (Liang *et al.*, 2019; Kusakabe *et al.*, 2020; Montiel *et al.*, 2021), while *Rhizobium* sp. NGR234 induces infection threads similar to *M. loti* (Schumpp *et al.*, 2009) and *R. tropici* CIAT899 induces infection threads in a manner that is dependent on the host *Lotus* species (Ayala-García *et al.*, 2022). In the present study, *Rhizobium* sp. Chiba-1 formed infection threads on *L. burttii*, while no infection thread was observed on *L. japonicus* MG051, MG063, or MG080, which formed mature like-nodules (Fig. 1 and 5, Table 1). These results showed that the invasion tactics by *Rhizobium* sp. Chiba-1 varied depending on the host plant. *S. fredii* HH103 is known to employ the NopC effector as a switch between intercellular invasion and infection thread formation in *L. burttii* (Jiménez-Guerrero *et al.*, 2020). However, neither a type III secretion system nor NopC effector genes were present in the genome of *Rhizobium* sp. Chiba-1. These results showed that the ability of *Rhizobium* sp. Chiba-1 to switch between intracellular and extracellular invasion pathways is dependent on as-yet-unidentified activities; therefore, the interactions between *Rhizobium* sp. Chiba-1 and *Lotus* that contribute to this host specificity warrant further study. Furthermore, *Rhizobium* sp. strain Chiba-1 formed two different infection threads in *L. burttii*. Long thin infection threads were more likely to be induced in the absence of typical root hair curling, while short thick infected threads showed root hair curling (Fig. 5). These differences in infection thread formation are expected to occur in association with unusual intracellular infections or general infection threads reaching into the nodules, and, thus, further studies are needed to identify the type of infected thread that reaches into nodules.

Based on previous findings suggesting that differences in the attachment behaviors of *Rhizobium* sp. IRBG74 and *M. loti* R7A to the root surface of *L. japonicus* Gifu affect the infection process (Montiel *et al.*, 2021; Quilbé *et al.*, 2022), we examined the attachment behavior of *Rhizobium* sp. Chiba-1 on both *L. japonicus* Gifu and *L. burttii* (Fig. 5A and B). The differences observed in rhizobial attachment behavior may contribute to the inability of *Rhizobium* sp. Chiba-1 to complement the symbiosis of the *M. loti ΔnodAC* mutant in co-inoculation experiments.

*Cbp1* expression was previously shown to be induced by Nod factors through a transcription factor such as CYCLOPS (Webb *et al.*, 2000; Gong *et al.*, 2022). In the present study, *Rhizobium* sp. Chiba-1 induced *Cbp1* expression in *L. japonicus* Gifu 3 days after the inoculation, similar to *M. loti* (Fig. 4), suggesting that Nod factors produced by *Rhizobium* sp. Chiba-1 were recognized by *L. japonicus* Gifu. In contrast, *Nin* expression was induced and fine-tuned by the complex involvement of several proteins and phytohormones, such as NSP1, NSP2, CYCLOPS, IPN2, LAN, GA, and CK (Singh *et al.*, 2014; Liu *et al.*, 2019; Suzaki *et al.*, 2019; Xiao *et al.*, 2020; Akamatsu *et al.*, 2021, 2022). *Rhizobium* sp. Chiba-1, unlike *M. loti*, was unable to induce *Nin* expression 3 days after the inoculation (Fig. 4), indicating that it may not be entirely capable of triggering the activation of these proteins. This variation in how the genes needed for symbiosis are expressed when *Rhizobium* sp. Chiba-1 is present may be directly connected to host specificity.

*Rhizobium* sp. Chiba-1, a unique *Rhizobium* that may symbiotically coexist with *Lotus* species, was successfully isolated in the present study. It differs from other known rhizobia in the host range and different infection processes between *Lotus* species. However, the mole­cular mechanisms underlying host specificity and the host range between *Rhizobium* sp. Chiba-1 and *Lotus* species remain unclear. Molecular genetic tools and bioresources from *Rhizobium* sp. Chiba-1 and *Lotus* species need to be used in the future to clarify signal mole­cular cues and perceptions, which are two functions connected to host specificity.

## Conclusion

We obtained the new *Rhizobium* sp. Chiba-1 strain that developed mature-like nodules on *L. burttii* and several *L. japonicus* wild-type accessions, but not on *L. japonicus* Gifu, the model accession, which provides novel insights into host specialization and the host range in symbiotic processes. Furthermore, we discovered that the epidermal infection process of *Rhizobium* sp. Chiba-1 was characterized by the production of infection threads in root hairs in a manner that was dependent on the host plant.

## Citation

Chiba, Y., Sasaki, M., Masuda, S., Shibata, A., Shirasu, K., and Kawaharada, Y. (2023) A Novel *Rhizobium* sp. Chiba-1 Strain Exhibits a Host Range for Nodule Symbiosis in *Lotus* Species. *Microbes Environ ***38**: ME23056.

https://doi.org/10.1264/jsme2.ME23056

## Supplementary Material

Supplementary Material

## Figures and Tables

**Table 1.** Nodule phenotypes in *Lotus* species inoculated with *Rhizobium* sp. Chiba-1.

		*n*	No. of plants forming mature-like nodules, including green nodules	% plants forming mature-like nodules, including green nodules	Ave. no. of mature-like nodules, including green nodules, per plant	Infection thread formation (11 dpi)
*L. japonicus*	Gifu	17	0	0	0±0	–
	MG20	18	0	0	0±0	nt
	MG008	20	5	25	0.4±0.2	–
	MG018	30	3	10	0.1±0.1	–
	MG022	18	1	6	0.1±0.1	–
	MG048	24	5	21	0.2±0.1	–
	MG051	21	20	95	3.6±0.4	–
	MG056	21	12	57	2.3±0.6	–
	MG063	30	26	87	5.8±0.7	–
	MG064	27	0	0	0±0	–
	MG073	20	1	5	0.1±0.1	–
	MG080	20	18	90	5.7±0.7	–
	MG098	8	0	0	0±0	–
	MG100	21	2	10	0.1±0.1	–
	MG111	30	4	13	0.3±0.2	–
	MG113	30	16	53	1.0±0.3	–
	MG115	30	1	3	0.1±0.1	–
	MG119	30	23	77	1.6±0.3	–
	MG122	29	2	7	0.1±0.1	–
	MG132	30	5	17	0.2±0.1	–
*L. burttii*		13	13	100	4.6±0.6	+
*L. filicaulis*		6	0	0	0±0	nt
*L. krylovii* cv China		10	0	0	0±0	nt
*L. krylovii* cv. Kazakhstan		10	0	0	0±0	nt

The nodulation phenotype 35 days after the inoculation “nt” is not observed
