# Supplementary material for: A Novel Rhizobium sp. Chiba-1 Strain Exhibits a Host Range for Nodule Symbiosis in Lotus Species
Source: Microbes Environ. 2023 Dec 1;38(4):ME23056. doi: 10.1264/jsme2.ME23056 (PMC10728632; doi:10.1264/jsme2.ME23056)
Supplement: Supplementary file 1 — Supplementary Material [file 38_23056_s1.pdf]

Supplemental Table 1. Homology identity in each gene between *R. leguminosarum* Norway and *Rhizobium* sp. Chiba-1.

Supplemental Figure 1. Nodule phenotypes in *Lotus* species.

Each *Lotus* species were 35 days after inoculation with *Rhizobium* sp. Chiba-1. Red arrows indicate mature-like nodules. Scale bars indicate 1 cm.

Supplemental Figure 2. *Trifolium repens* nodulation.

(**A**) The right *T. repens* formed *a* mature nodule 35 days after inoculation with *Rhizobium* sp. Chiba-1. The left *T. repens did not form* any nodules for control 35 days after inoculation with H_2O. The scale bar indicates 1 cm. Arrows indicate mature nodules. (**B**) Average of each nodule type number formed over time after inoculation (DAI) with *Rhizobium* sp. Chiba-1 in *T. repens*. *n* = 7. Error bars indicate SE.

Supplemental Table 1. Homology identity in each gene between *R. leguminosarum* Norway and *Rhizobium* sp. Chiba-1

	Gene	gene ID	Identiy to *R. leguminosarum* Norway	
			Nucleotide	Proitein
housekeeping	*16S rRNA*	Rchiba_05010 16S rRNA (uracil(1498)-N(3))-methyltransferase	99%	-
	atpD	Rchiba_41760 F0F1 ATP synthase subunit beta	93%	97%
	recA	Rchiba_23600 recombinase RecA	97%	99%
	dnaK	Rchiba_46700 molecular chaperone DnaK	99%	99%
	rpoB	Rchiba_16320 DNA-directed RNA polymerase subunit beta	98%	99%
Nod factor synthesis	*nodA*	Rchiba_73750 nodulation N-acyltransferase NodA	76%	81%
	nodB	manual gene annotation	73%	74%
	nodC	Rchiba_73740 chitooligosaccharide synthase NodC	74%	75%
	nodD	Rchiba_71900 transcriptional regulator NodD2	75%	77%
		Rchiba_73760 transcriptional regulator NodD2	75%	78%
		Rchiba_76200 transcriptional regulator NodD2	75%	78%
		Rchiba_77400 transcriptional regulator NodD2	76%	78%
		Rchiba_75640 hypothetical protein	75%	79%
	nodE	Rchiba_73780 host-specificity nodulation protein HsnB	75%	77%
	nodF	Rchiba_73770 host-specificity nodulation protein HsnA	77%	77%
	nodI	Rchiba_73730 nodulation factor ABC transporter ATP-binding protein NodI	77%	79%
	nodJ	Rchiba_73720 ABC transporter permease	76%	86%
	nodL	Rchiba_73800 nodulation O-acetyltransferase NodL	77%	85%
	nodM	Rchiba_73810 glutamine--fructose-6-phosphate transaminase (isomerizing)	74%	76%
	nodN	Rchiba_73820 nodulation protein NodN	82%	73%
Nitrogen fixation and cytochrome oxidase	*fixA*	Rchiba_73610 electron transfer flavoprotein subunit beta/FixA family protein	69%	73%
	fixB	Rchiba_73620 electron transfer flavoprotein subunit alpha/FixB family protein	70%	74%
	fixC	Rchiba_73630 FAD-dependent oxidoreductase	75%	77%
	fixG	Rchiba_73300 cytochrome c oxidase accessory protein CcoG	93%	92%
	fixH	Rchiba_73310 FixH family protein	98%	96%
	fixI	Rchiba_73320 cation-translocating P-type ATPase	94%	95%
	fixN	Rchiba_73260 cytochrome-c oxidase, cbb3-type subunit I	95%	97%
	fixO	Rchiba_73270 cytochrome-c oxidase, cbb3-type subunit II	98%	99%
	fixP	Rchiba_73290 cytochrome-c oxidase, cbb3-type subunit III	98%	98%
	fixQ	Rchiba_73280 CcoQ/FixQ family Cbb3-type cytochrome c oxidase assembly chaperone	97%	100%
	fixS	Rchiba_73330 cbb3-type cytochrome oxidase assembly protein CcoS	96%	98%
	fixX	Rchiba_73640 ferredoxin family protein	76%	76%
Nitorogenae	*nifA*	Rchiba_73650 hypothetical protein	69%	65%
	nifB	Rchiba_73660 nitrogenase cofactor biosynthesis protein NifB	72%	67%
	nifD	Rchiba_73590 nitrogenase molybdenum-iron protein alpha chain	78%	82%
	nifE	Rchiba_73570 nitrogenase iron-molybdenum cofactor biosynthesis protein NifE	77%	75%
	nifH	Rchiba_73600 nitrogenase iron protein	81%	92%
	nifK	Rchiba_73580 nitrogenase molybdenum-iron protein subunit bet	78%	85%
	nifN	Rchiba_73560 nitrogenase iron-molybdenum cofactor biosynthesis protein NifN	71%	69%
Type IV secreation system	*virB1*	Rchiba_54310 lytic transglycosylase domain-containing protein	85%	82%
	virB2	Rchiba_54320 TrbC/VirB2 family protein	93%	97%
	virB3	Rchiba_54330 type IV secretion system protein VirB3	88%	89%
	virB4	Rchiba_54340 VirB4 family type IV secretion system protein	92%	95%
	virB5	Rchiba_54350 P-type DNA transfer protein VirB5	91%	91%
	virB6	Rchiba_54370 type IV secretion system protein	92%	96%
	virB8	Rchiba_54390 virB8 family protein	90%	93%
	virB9	Rchiba_54400 P-type conjugative transfer protein VirB9	91%	95%
	virB10	Rchiba_54410 type IV secretion system protein VirB10	90%	93%
	virB11	Rchiba_54420 P-type DNA transfer ATPase VirB11	90%	95%
	virD4	Rchiba_75530 type IV secretory system conjugative DNA transfer family protein	48%	31%
Exopolysaccharide synthesis and export	*pssW*	Rchiba_34470 endo-1,4-beta-xylanase	98%	99%
	pssS	Rchiba_34480 glycosyltransferase	98%	99%
	pssR	Rchiba_34490 hypothetical protein	97%	98%
	pssM	Rchiba_34500 polysaccharide pyruvyl transferase family protein	91%	96%
	pssL	Rchiba_34510 lipopolysaccharide biosynthesis protein	95%	98%
	pssJ	Rchiba_34530 galactosyl transferase	94%	97%
	pssG	not sequenceed		
	pssH	not sequenceed		
	pssI	Rchiba_34540 hypothetical protein	88%	92%
	pssF	Rchiba_34550 hypothetical protein	91%	95%
	pssC	Rchiba_34560 hypothetical protein	97%	97%
	pssD	Rchiba_34580 PssD/Cps14F family polysaccharide biosynthesis glycosyltransferase	97%	99%
	pssE	Rchiba_34590 PssE/Cps14G family polysaccharide biosynthesis glycosyltransferase	98%	99%
	pssP	Rchiba_34660 polysaccharide biosynthesis tyrosine autokinase	97%	99%
	pssN	Rchiba_34680 polysaccharide biosynthesis/export family protein	96%	99%
	pssT	Rchiba_34690 O-antigen ligase family protein	92%	92%
	pssA	Rchiba_35620 sugar transferase	97%	98%

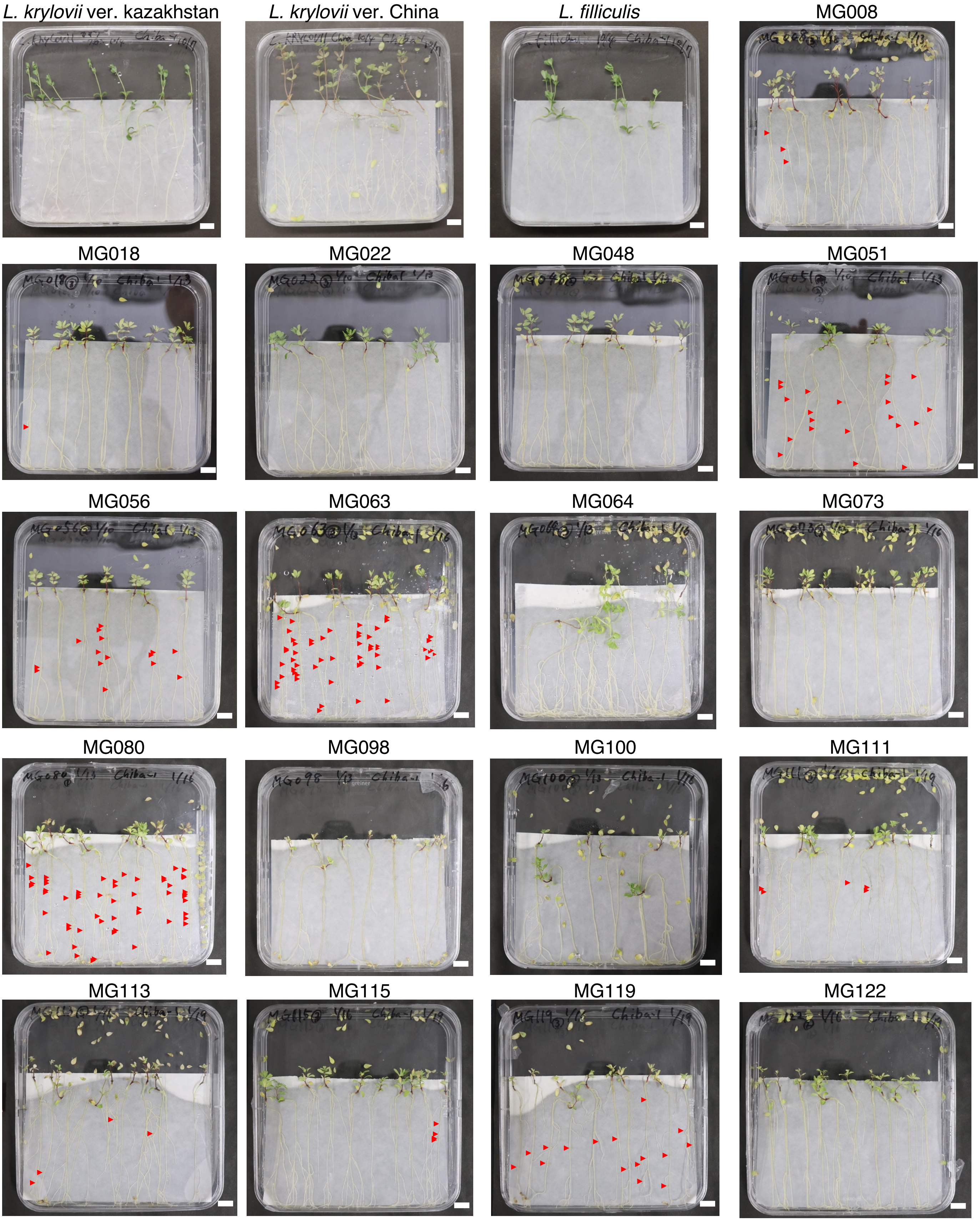

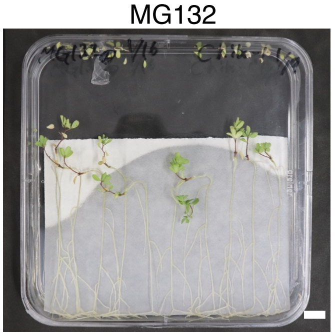

Supplemental Figure 1. Nodulation phenotypes in *Lotus* species.

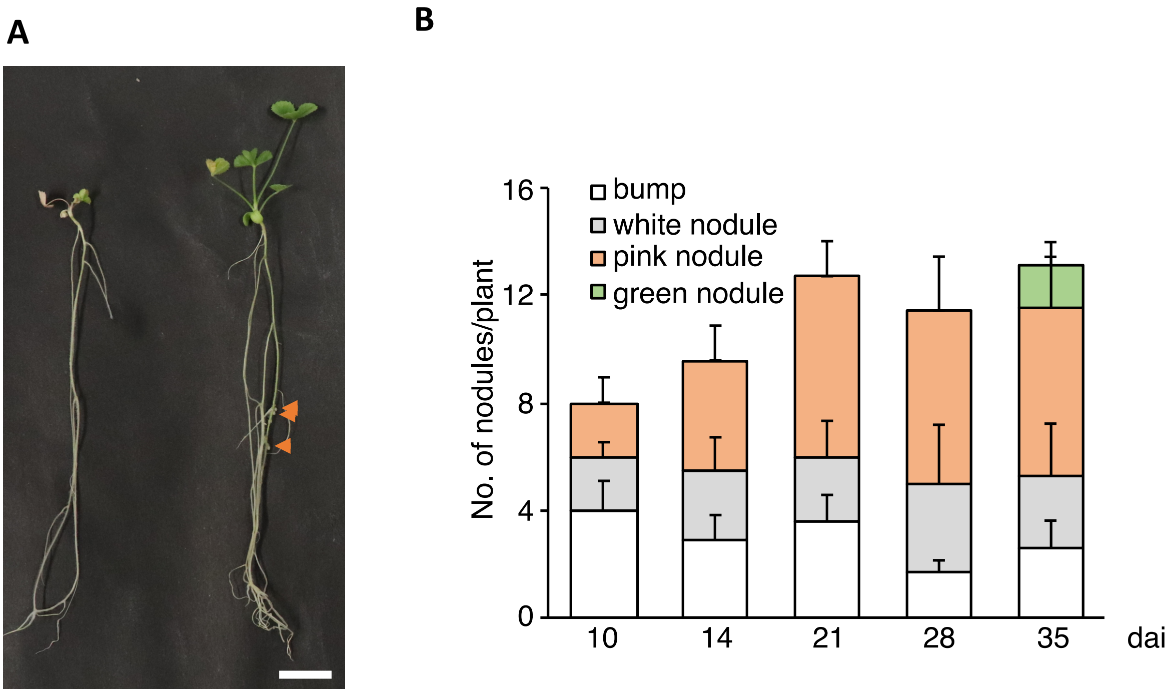

Supplemental Figure 2. *Trifolium repens* nodulation.
